# Characterization of Genetic and Agromorphological Variation in 30 Soybean (*Glycine max*) Accessions in Northern and Southern Benin

**DOI:** 10.1155/sci5/3449081

**Published:** 2025-02-24

**Authors:** Vincent Ezin, Fatimata Bachabi, Florida Corolle Mintodè Dubogan, Wassiou Ahanchede, Gazali B. T. A. Sanni, Mohamed Salim Moussa, Adam Ahanchede

**Affiliations:** ^1^Department of Crop Production, Faculty of Agricultural Sciences, University of Abomey-Calavi, 01 BP 526, Cotonou, Benin; ^2^Africa Rice Center, Germplasm Health Unit, 01 BP 2551, Bouake 01, Côte d'Ivoire

**Keywords:** genetic variability, *Glycine max*, legume, multienvironmental trials, phenotypic traits

## Abstract

Soybean is a highly nutritious and versatile food that is of great importance in world trade. It is a cash crop for farmers in Benin, grown almost everywhere. However, soybean production faces several challenges, including low yields and disruptions due to weather conditions. Improving soybean production could enhance food security for the population and increase farmers' incomes. A study was conducted to assess the genetic and agromorphological variability of 30 soybean accessions. The study was carried out in a three-repeat alpha lattice design at two different sites in the South of Benin (E1) and North (E2). Qualitative and quantitative traits were collected during the experiments. The qualitative characteristics showed great variability, except for cotyledon color and the presence of pubescence and petiole. The results also showed that Accessions TGm-1199, TGX 1910-14, and TGm-1588 had the best yields in E1, whereas TGX 1951-3F, TGX 1910-14F, and TGm-1253 were found with the highest yields in E2. The phenotypic coefficient of variation was higher than the genotypic coefficient of variation for all traits, and heritability in the broad sense ranged from 20% to 100%. The genetic parameters showed that selection programs can be effective for days to flowering, plant height, hundred-seed weight, seed thickness, seed length and width, days to harvest, pod length and width, and grain yield. The high genetic variability observed within soybean accessions indicates that genotypes could be selected and used as a crop improvement tool.

## 1. Background

Soybean, scientifically known as *Glycine max* (L.) Merr., is a fast-growing plant with high nutritional value and is one of the most important legume crops in the world. It is a rich source of oil (18%–21%), carbohydrates (26%–30%), and protein (40%–45%), which makes it a crucial component of human and animal diets and various industrial applications [1–3]. It is the most important legume in terms of production and marketing. The main producing countries are Brazil, the United States, Argentina, China, and India, which together account for 80% of global soybean production [4, 5]. According to the Food and Agriculture Organization (FAO), soybean production increased from 231 million tons in 2008 to 353 million tons in 2020. This growth was achieved by cultivating soybeans on 126 million hectares [6]. Although global soybean production has been growing consistently at a compound annual growth rate (CAGR) of 3.6% between 2008 and 2020, it has decreased to a CAGR of 2.42% between 2014 and 2020 according to FAOSTAT [7]. However, in 2021, global soybean production exceeded the estimated quantity and reached 388,098 metric tons as reported by FAOSTAT [5]. In Africa, there is little to no significant growth in the production of soybeans, despite their value, vital uses, and profits [8]. However, in recent times, soybean cultivation is gaining popularity in Africa, with the crop being the latest to be adopted by the continent. Africa's crop production is increasing by 6.84% annually, mainly due to expanded crop area rather than increased yield (YLD) [6, 9]. Africa's contribution to the global soybean industry is insignificant, with its producers supplying less than 1% of the world's soybeans. This is mainly due to the insistent YLD gap between smallholder producers and large-scale farmers, which is a common issue in both developing and developed countries [6, 9]. The underusage of up-to-date inputs in developing countries has led to a stagnant average soybean YLD of 1.1 t·ha^−1^ in SSA, which is much lower than the global average of 2.4 t·ha^−1^. Soybean production in Africa is mainly intense in South Africa (35%), followed by Nigeria (27%) and Uganda and Zambia (8.5%). Other countries such as Zimbabwe, Ghana, Sudan, Malawi, and Ethiopia have also seen significant growth in commercial cultivation [6, 8]. In recent years, soybean cultivation has gained commercial importance due to its demand for human consumption, livestock, poultry feed, and the agrifood industries.

Over the years, the production of soybeans in Benin has increased significantly from 57,000 tons in 2009 to 306,197.99 tons in 2022 at an average annual rate of 26.00% [5]. Despite the existence of a market outlet, commitment from private bodies to promote the sector, and the desire of producers to increase production, the soybean sector is still struggling to take off. Soybeans are mainly used for human consumption in the form of flour, cheese, and milk, and it is becoming more popular in the Beninese diet. Identified as an emerging commodity chain in the Strategic Plan for Agricultural Sector Development (PSDSA), soybeans are now a cash crop for the rural population [10] and are a promising commodity chain for Beninese agriculture. However, soybean production faces several challenges. Even when the best cultivation practices are used, YLDs remain below potential. Soybean cultivation faces problems such as the dehiscence of pods at maturity, disturbances due to climatic hazards—especially high temperatures—and the loss of germination capacity [11]. Genetic improvement can play a critical role in ensuring that soybean remains the leading crop. The development of high-yielding varieties that can resist climatic hazards depends on the extent of genetic variability available within the population. Therefore, it is essential to have a better understanding of the genetic characteristics and parameters for a thorough knowledge of germplasm and its manipulation in any crop improvement program. This study aims to assess the existing diversity within soybean accessions from Benin and Nigeria, identify the elites that can be exploited in varietal selection programs, and create a basic collection for various uses. The general objective of this study is to analyze the genetic diversity within soybean (*Glycine max*) accessions in Benin and Nigeria. In particular, the study objectives are to (i) assess the agromorphological variability of soybean accessions and (ii) study the genetic variability within soybean accessions.

## 2. Materials and Methods

### 2.1. Description of Two Experimental Sites

The study took place in Benin at two different sites. The first site was in the southern region at IITA Benin (E1). The second site was in the northern region in the commune of Basslila, in the village of Pénéssoulou (E2) (Table 1).

### 2.2. Experimental Material

Thirty soybean accessions, 22 from IITA Ibadan and 8 from IITA Benin, were used in this study (Table 2).

### 2.3. Experimental Setup and Crop Management

The 30 accessions were evaluated in an alpha-lattice design with three replicates. The plot consisted of three rows, each representing a replication and separated from each other by 2 m. The blocks were separated from each other by 1 m, and the plot units of each block were separated by 2 m.

The blocks were separated by 1 m for the first trial and 0.5 m for the second trial. Each replication contained 6 blocks, each consisting of 5 plot units measuring 1.20 × 1.50 m, resulting in a total of 18 blocks and 90 plot units. The spacing of the seedlings was 50 × 40 cm on each plot unit. Sowing was performed on June 2 for the first trial and on July 4 for the second trial at a rate of three seeds per hole and a depth of 2 cm. Resowing was carried out 10 days later, and after germination, only the strongest seedling in each hole was retained. Weeds were controlled manually and chemically using K-optimal at a dose of 50 mL per 15 L of water. Phytosanitary treatment was carried out every fortnight until the pods reached maturity. The pods were harvested when they reached harvestable maturity.

### 2.4. Parameters Measured

In the experiments, we measured a total of 35 variables, consisting of 17 quantitative and 18 qualitative variables (Table 3). In particular, we measured and observed the three best plants located in the center of each experimental unit. For this purpose, we used the list of descriptors that UPOV defined for soybeans in 1998 to ensure accurate measurements and observations.

#### 2.4.1. Quantitative Parameters

The plant population per hectare was estimated as follows:

(1) $$\begin{array}{c} \text{Pp}=\frac{10000\;m^{2}}{\left(\text{the product of spacing}\right)},\\ {\text{LS⁣mm}}^{2}=\text{TLL}*\text{TLW},\\ {\text{SSi⁣mm}}^{3}=\text{SL}*\text{SWi}*\text{ST}. \end{array}$$

#### 2.4.2. Qualitative Parameters

The 18 qualitative variables are shown in Table 4.

### 2.5. Statistical Analysis of Data

#### 2.5.1. Analysis of Variance (ANOVA)

The grain YLD data obtained from two different sites were analyzed using the R software. The ANOVA and combined analysis of variance (CANOVA) were employed to determine the significance of genotype, environment, and genotype-by-environment interaction. The replications at each site were considered as random effects, whereas genotypes were regarded as fixed effects. The following model was used for the CANOVA:

(2) $$\begin{array}{c} Y_{ijkl}=\mu +G_{i}+E_{j}+R_{k\left(j\right)}+B_{l\left(Jk\right)}+{GE}_{ij}+\varepsilon_{ijk}, \end{array}$$

where *Y*_*ijkl*_ is the response of the *i*^th^ genotype in the *j*^th^ environment and *k*^th^ replication in the environment and *l*^th^ block in the replication; *μ* is the overall mean, *G*_*i*_ is the genotypic effect *i*; *E*_*j*_ is the environment effect *j*; *R*_*k*(*j*)_ is the replication in environment effect *k*; *B*_*l*(*Jk*)_ is the block in replication effect *l*; *GE*_*ij*_ is the genotype × environment interaction effect; and *ε*_*ijk*_ is the random error.

#### 2.5.2. Principal Component Analysis (PCA) and Hierarchical Clustering Analysis (HCA)

To identify the types of correlation that exist between quantitative variables, we conducted Pearson's correlation. Then, we performed PCA and HCA in the R program on the quantitative variables. We used the “FactoMineR” package to group accessions into distinct categories. Finally, we visualized the dendrogram generated by the “FactoMineR” package using the “factoextra” package via the HCPC function.

### 2.6. Genotype Performance

A statistical analysis was also conducted to analyze the performance of the accessions and identify the differences in their performance based on different environmental conditions. The accessions were ranked based on their performance for each trait, from the best to the worst. The study involved analyzing the traits of the different accessions in pairs, simultaneously in both environments, to determine how they behave in one environment compared with the other.

## 3. Result

### 3.1. Description of Variables

Table 5 displays various quantitative variables, with the highest coefficient of variation values found for YLD (52.5053), total seed weight (TSW) (51.3317), number of pods per plant (NPP) (49.8069), and plant size at harvest (49.4709). YLD varied from 0.01 to 5.3017 tons per hectare, with an average of 0.564 tons per hectare. The NPP ranged from 9 to 636, with an average of 158.12 pods per plant. Flowering date ranged from 30 to 63 days, and the harvest date (DH) ranged from 78 to 148 days. The maximum plant height at flowering was 60.8 cm, and the maximum height at harvest was 118.5 cm or 1.18 m.

### 3.2. ANOVA

An ANOVA was conducted to assess the impact of different environments on various quantitative variables. The study revealed significant differences between environments, replicates, blocks, and genotypes, as well as between environment–replicate, replicate–block, and block–genotype interactions across most traits (Table 6). The genotypes exhibited a highly significant difference (*p* < 0.001). At the block level, all variables, except days to flowering (DF), TSW, and YLD, showed very significant (*p* < 0.01) and highly significant (*p* < 0.001) effects. Regarding the variance between replicates, only seed width (SWi) showed a highly significant difference (*p* < 0.001). In contrast, the terminal leaflet length (TLL) and terminal leaflet width (TLW) showed a highly significant difference (*p* < 0.01), and variables such as DH, pod length (PL), seed thickness (ST), TSW, and YLD showed a significant difference (*p* < 0.05). All variables exhibited highly significant (*p* < 0.001) and very highly significant differences across different environments, except the number of leaflets (NL), which showed no difference (Table 6).

A highly significant difference (*p* < 0.001) was observed in all variables at the genotype × environment interaction level. These variables help us to better understand the effect of the environment and the difference between the varieties.

Environmental × repetition interaction showed a highly significant difference (*p* < 0.01) in the NPP and pod width (PW) and a significant difference (*p* < 0.05) in ST.

Similarly, the replication × blocks and blocks × genotypes interaction had significant effects on DF, DH, NPP, PL and PW, number of seeds per pod (NSP), seed length (SL), ST, and hundred-seed weight (HSW).

### 3.3. Morphological Traits

Upon comparing the NL of different accessions, it was noted that there exists a significant difference between varieties regarding the NL (Table 7). The Varieties V22 and V24 exhibited the highest NL (5). The longest terminal leaflet was observed in Accessions V1, V2, V8, V12, V14, and V15, measuring 9 cm. However, Accession V6 had the widest terminal leaflet, measuring 7.22 cm. During the onset of the flowering phase (plant height at R1 [PHR1]) and the maturity phase (PHR8), a highly significant difference in plant height was observed. Accessions V24, V1, and V28 were the tallest during the flowering phase, measuring 41.56, 40.82, and 40 cm, respectively. However, during maturity, Accessions V2 and V13 were the tallest, measuring 66.95 and 63.66 cm, respectively. The shortest plants were recorded in Accessions V20, V11, and V6, which measured 15.55, 17.26, and 23.61 cm, respectively. Accessions with longer flowering dates were V28 and V4, which flowered at 59 and 56 days, respectively. In contrast, Accession V20 had the earliest flowering date, which occurred only 30 days after planting (Table 7).

### 3.4. Analysis of YLD Parameters

Based on the comparison of accession variable averages, it is evident that there is a highly significant difference between accessions for YLD parameters (Table 8). Accession V1 records the highest NPP (273), followed by Accessions V3 (230) and V24 (261). Analysis of Table 8 reveals that there was a significant difference between Accessions V6, V8, and V2 and other accessions for PL. Accessions V6 (46 cm), V8 (44 cm), and V2 (44 cm) exhibited very high PLs, whereas the lowest PLs were recorded in V9 (27 cm) and V28 (27 cm). However, there was hardly any significant difference between the different soybean accessions in terms of PW, NSP, SL, SWi, and ST.

Furthermore, a highly significant difference has been observed between accessions regarding YLD per plot unit (TSW) and average seed YLD. The accession with the highest YLD was V2 (596 g), followed by V8 (161 g) and V1 (114 g). Accessions V20 (17 g) and V13 (30 g) recorded the lowest YLDs per plot unit (Table 8).

### 3.5. PCA

**Figure 1:**
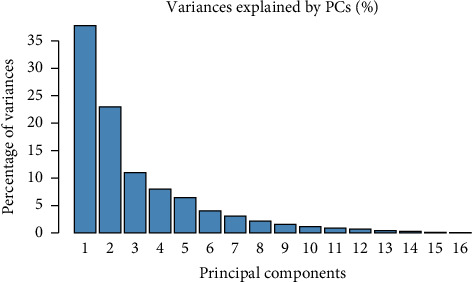
Breakdown of the total information contained in each axis.

**Figure 2:**
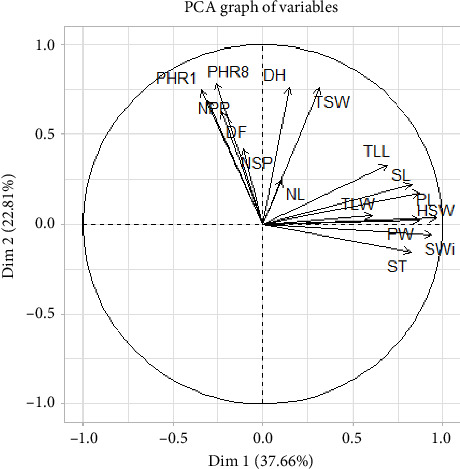
Correlation circle of variables with the two main axes.

PCA was conducted on the 16 quantitative variables, across two environments (Figure 1). The analysis revealed that the first five components had eigenvalues greater than 1.00, as shown in Table 9. The first two dimensions of the PCA explained 60.46% of the total data inertia, with each dimension accounting for 37.66% and 22.80%, respectively (Table 9). This implies that this PCA represents 60.46% of the variability in the clouds of variables or individuals (Figure 1). As this percentage is quite high, it can be concluded that the first two dimensions (Dim1 and Dim2) encompass the majority of all active variables (Figure 2).

The first principal component (PC1) showed a positive correlation with the variables SL (0.82), PL (0.87), HSW (0.96), PW (0.86), SWi (0.94), ST (0.82), TLL (0.69), and TLW (0.60). Among these variables, SL, PL, HSW, PW, SWi, and ST exhibited high correlation coefficients (Table 10).

The second principal component (PC2) was positively correlated with PHR1 (0.74), PHR8 (0.78), TSW (0.75), DH (0.76) (high correlation), DF (0.62), NPP (0.68) (moderate correlation), and NSP (0.42) and NL (0.24) (low correlation). This component was negatively correlated with SWi (−0.05) and ST (−0.15) (Table 10).

**Figure 3:**
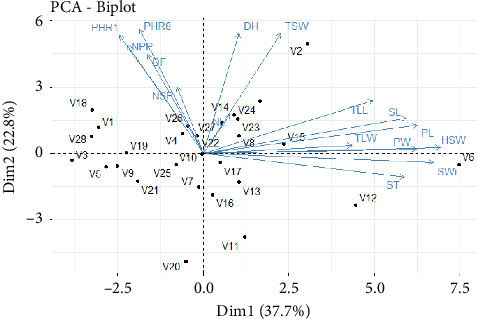
Biplot PCA of the average of the two environments.

Component 1 or Axis 1 indicates that V12, V6, and V15 accessions generally had long and wide leaflets (9.02–12.95 cm and 4.71–7.12 cm, respectively), the longest and widest pods (37.22–46.2 mm and 5.75–10.52 mm, respectively) with the greatest thickness (4.93–5.65 mm), and large seeds with the highest HSWs (17.14–21.29 g) (Figure 3). The highest values of plant size at flowering and maturity (30.60–42.25 cm and 34.69–47.28 cm, respectively), vegetative cycle (110–113 days), flowering date (50–56 days), NPP (106–256 pods), and NSP (2.5–3 seeds) were observed in V18, V1, V4, V26, and V19 accessions. However, the lowest values were found in the other accessions (Figure 3).

On Component 2 or Axis 2, V22, V27, and V14 accessions had a very high DH, with a high NSP and a high plant height, as well as longer flowering dates and high TSW. This resulted in a high YLD per plot (Figure 3). However, on Axis 2, to a lesser extent, V24, V26, V4, and V2 accessions exhibited all the above characteristics of V22 accession. These accessions yielded the highest total weights and consequently the best YLDs.

### 3.6. Hierarchical Classification of Soybean Accessions

**Figure 4:**
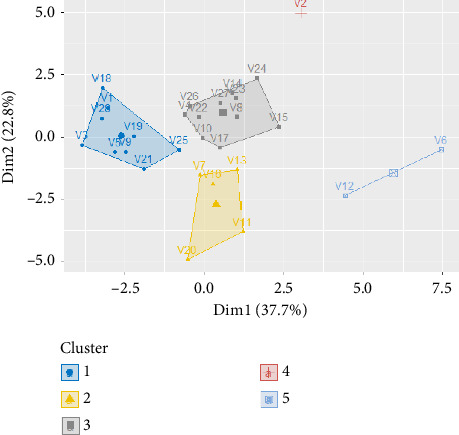
Soybean accession classification.

The ascending hierarchical classification revealed five different groups within the soybean accessions (Figure 4 and Table 11). The first class is made up of Accessions V18, V1, V28, V3, V5, V9, V19, V21, and V25. Accessions in this class had late flowering (0.776), the highest NPP (1.63), the highest plant size at flowering (1.53), and moderately high plant size at maturity (1) (Table 11). The second class is made up of Accessions V7, V13, V16, V1, and V20, which on average had moderately high ST (0.999) and SWi (0.821); the remaining characters were of low values. The third class comprises V4, V8, V10, V14, V15, V17, V22, V23, V24, V26, V27, and V28. This class is an average class in terms of the various quantitative variables, as almost all these variables were generally of average value (good values), except for NPP, which is the only one with a low value (−0.102). With regard to the vegetative cycle, ST, and SWi variables, Class 3 occupies the second position, with values of 2.02, 1.81, and 1.36, respectively. As for Class 4, only the highest-yielding V2 accession is found here, as its TSW is much higher than all the other classes, with a modality of 4.51. These other variables are also interesting, with the exception of ST and TLW, which had negative values.

The last class is made up of two accessions (V6 and V12). Both had higher values than the other classes for YLD and YLD components (PL, PW, SL, ST, and SWi) and leaflet (TLL and TLW) parameters. The HSW of the latter class also had the highest value (3.23). In terms of TSW, it ranked third with a value of −0.076. It also had the lowest values for flowering time DF (−2.02), vegetative cycle (−0.906), NPP (−1.03), and plant size at flowering and harvest (PHR1 and PHR8).

Tables 12 and 13 revealed the performance of accessions in each environment based on quantitative traits measured.

The performance of genotypes and their environments using descriptive analysis is presented in Table 14.

### 3.7. Genetic Variability

Table 15 provides estimates of genotypic and phenotypic variance, genotypic coefficient of variation (GCV) and phenotypic coefficient of variation (PCV), broad heritability, and genetic gain for different traits. The genotypic variance for the traits ranged from 0.14 for the number of grains per pod to 16,353.83 for total grain weight, whereas the phenotypic variance ranged from 0.24 for the number of grains per pod to 23,782.09 for total grain weight. The highest values of genotypic and phenotypic variance were observed for total kernel weight, NPP, plant height at maturity, and DH. However, the lowest variances were found for NL, TLW, PW, number of grains per pod, SL, SWi, and ST.

Table 15 presents data indicating that all studied parameters have higher PCV values than GCV values. It was observed that the parameters, such as flowering date and DH, showed a single value for both coefficients. When there is a large difference between GCV and PCV values, it is an indication that environmental factors dominate trait expression. This holds true for the parameters, including TLL, TLW, NPP, plant height at the start of flowering, number of primary branches at maturity, and NSP. However, a small difference between coefficients demonstrates the influence of genetic factors. This is the case for the parameters—NL, hundred-grain weight, SL, SL and ST, plant height at maturity, and total grain weight. Total kernel weight showed the highest GCV and PCV values, indicating a high degree of variability for this trait.

The following parameters have high heritability—leaflet number (84.33%), flowering date (100%), DH (100%), grain length (85.95%), and hundred-kernel weight (89.84%). Plant height at maturity (63.20%), PL (74.60%), SWi (78.29%), ST (78.23%), and total kernel weight (68.77%) have moderately high heritability (Table 15). Heritability is moderate for parameters such as TLW (57.32%), plant height at the start of flowering (55.56%), PW (55.93%), NSP (56.72%), and number of primary branches at maturity (55.62%). TLL (37.98%) and NPP (20.25%) have low heritability. High heritability, when accompanied by high genetic gain, indicates that selection can be effectively applied to the trait concerned. This is the case for plant height at maturity, hundred-kernel weight, and total kernel weight.

### 3.8. Pearson's Correlation Among Soybean Traits

**Figure 5:**
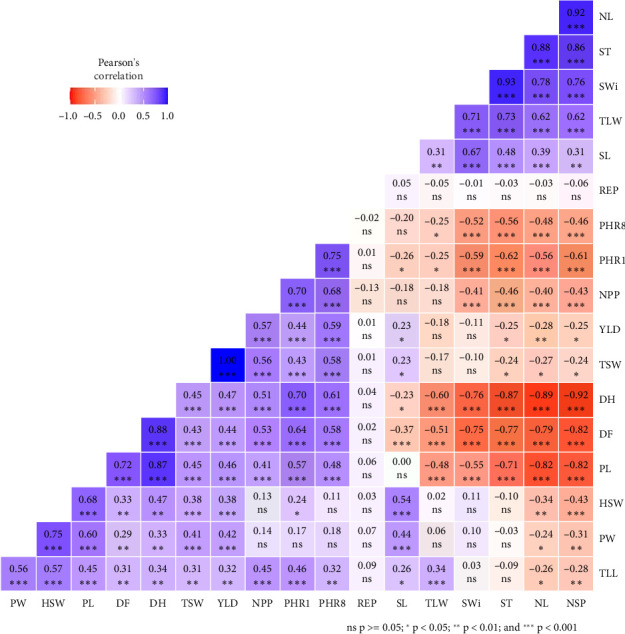
Pearson's correlations among the phenological, morphological, and agronomic traits.

The Pearson correlation matrix shows correlation coefficients ranging from −1.0 to 1.0, with color coding (blue for positive correlations and red for negative correlations) and significance levels indicated by asterisks (Figure 5). The correlation analysis of soybean YLD and its components reveals significant relationships across various traits. NPP shows a positive and significant correlation with YLD (*r* = 0.57⁣^∗∗∗^). YLD also demonstrates positive and significant correlations with both harvest date (DH) (*r* = 0.47⁣^∗∗∗^) and DF (*r* = 0.44⁣^∗∗∗^). DH exhibits strong negative correlations with the NSP (*r* = −0.92⁣^∗∗∗^), NL (*r* = −0.89⁣^∗∗∗^), and SWi (*r* = −0.76⁣^∗∗∗^).

The NL demonstrates positive and significant correlations with SWi (*r* = 0.78⁣^∗∗∗^) and ST (*r* = 0.88⁣^∗∗∗^). PHR1 shows a positive and significant correlation with plant height at R8 (PHR8) (*r* = 0.75⁣^∗∗∗^) and negative correlations with SWi (*r* = −0.59⁣^∗∗∗^) and NSP (*r* = −0.61⁣^∗∗∗^). TLW exhibits a positive and significant correlation with the NL (*r* = 0.62⁣^∗∗∗^) and ST (*r* = 0.73⁣^∗∗∗^) (Figure 5).

HSW shows a significant and positive correlation with PW (*r* = 0.75⁣^∗∗∗^) and a significant and positive correlation with PL (*r* = 0.68⁣^∗∗∗^). TLL demonstrates significant and positive correlations with plant weight (PW) (*r* = 0.56⁣^∗∗∗^) and PL (*r* = 0.45⁣^∗∗∗^) while showing negative correlations with the NSP (*r* = −0.28⁣^∗^) (Figure 5).

### 3.9. Analysis of Qualitative Variables

#### 3.9.1. Characteristics of the Vegetative Stage of the Plant

**Figure 6:**
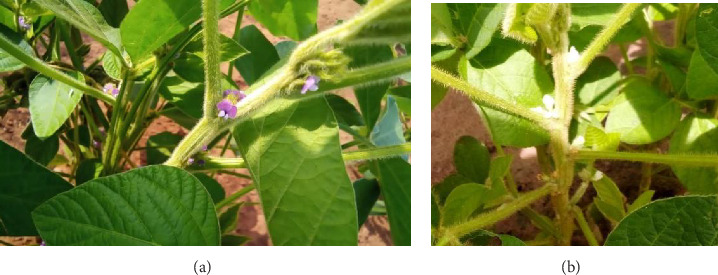
(a) Purple flowers and (b) white flowers.

It has been observed that the accessions studied showed a certain degree of variability, except for the color of the cotyledon, which is green in all accessions, as well as for the presence of petioles and pubescence (Figure 6). Of the 18 morphological descriptors listed in Table 4, the majority of accessions had a purple hypocotyl (77.78%) and normal density pubescence (40.74%); however, some had green hypocotyls (22.22%) and varying degrees of pubescence density, ranging from dense (18.52%) to sparse (18.52%). The color of pubescence also varied, with most accessions having light brown pubescence (51.85%), whereas others had dark brown (22.22%) or gray (25.93%) pubescence (Table 16). The type of pubescence varied as well, ranging from erect (29.63%) to curly (3.70%), including semi-appressed (44.44%) and appressed (22.22%). For leaflet shape, the majority of accessions (55.56%) had broad oval leaflets, whereas 29.63% had narrow lanceolate leaflets and only 14.81% had intermediate-shaped leaflets. Most accessions (92.59%) had small-sized leaflets, whereas only 7.41% had medium-sized leaflets. The flower color analysis showed that 85.19% of the accessions had purple flowers and 14.81% had white flowers. For growth habit, 22.22% of the accessions had an erect habit, 22.22% had an erect to semierect habit, 48.15% had a semierect habit, and 7.41% had a semierect to horizontal habit. The majority of individuals (59.26%) had determinate growth, whereas the remaining 40.74% had indeterminate growth. Finally, at the beginning of the fruiting, 44.44% of the accessions were not subject to lodging, 33.33% were not very sensitive to lodging, 7.41% had a moderate level of lodging, 11.11% had a severe level of lodging, and 3.70% had a very severe level of lodging (Table 16).

#### 3.9.2. Characteristics of Pods and Seeds

**Figure 7:**
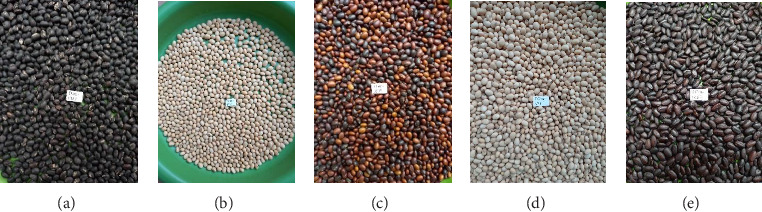
Black seeds (a), yellowish white (b), reddish brown (c), light brown (d), and imperfect black (e).

Of all the accessions studied, 59.26% had light brown pods, 37.04% had dark brown pods, and only 3.70% had black pods (Table 16; Figure 7). In terms of pod dehiscence, 48.15% of the population had poorly dehiscent pods, 40.74% had indehiscent pods, and 7.41% had very dehiscent pods (Table 16). Light brown is the dominant color of the seeds, with 74.07% of the accessions. However, four other colors were observed as well, including black (7.41%), imperfect black (3.70%), yellowish white (7.41%), and reddish brown (7.41%) (Table 16; Figure 7). There were five main colors noted for the hilum, with 14.81% of the accessions having a black hilum, 22.22% having a brown hilum, 18.52% having a light brown hilum, 7.40% having a gray hilum, and 37.04% having an imperfect black hilum. For integument, 48.15% of the seeds had a dull integument, 18.52% had a shiny integument, and the rest presented an intermediate phenotype. Table 16 shows the distribution frequencies of soybean qualitative traits.

## 4. Discussion

The evaluation of soybean accessions from Benin and Nigeria revealed the existing diversity among them based on qualitative and quantitative characteristics. Most of the qualitative characters studied showed variations in their frequencies of expression, ranging from 3.70% to 100%. Srinives et al. [12], Ramteke et al. [13], and Ezin, Gbemenou, and Ahanchede [14] also demonstrated that qualitative characters are stable over generations and less influenced by environmental conditions. Like the present study, they also reported high variability for the traits studied.

Pubescence is a crucial morphological character of soybean genotypes that contributes to resistance against biotic and abiotic stress. Morojele et al. [15] found in their study that all cultivars were pubescent, which is consistent with the results of the present study. Most of the leaflets of soybean plants were oval, and no large leaflets were observed during the experiment, which is in agreement with the findings of Morojele et al. [15]. The increase in leaf surface area leads to high levels of chlorophyll and a high rate of photosynthesis, which should be considered for improving soybean YLD.

Observations on lodging showed that most accessions were prone to lodging at different severity levels. Large plants with a high NPP, primary branches at maturity, and indeterminate growth were the most affected by lodging. When mature, most of the pods turned a light brown color. Ningsih, Zubaidah, and Kuswantoro [16] obtained the same result in their study. Pod dehiscence was evaluated, and more than half of the accessions studied had dehiscent pods, which is consistent with the findings of Agrawal et al. [17]. They stated that pod bursting is influenced by environmental factors such as dry climate, low air humidity, high temperatures, rapid temperature change, and genetic factors. Pod dehiscence at maturity is a real constraint in soybean cultivation, causing 34%–100% YLD loss depending on the level of severity. Selecting accessions resistant to pod shattering could contribute to improving YLD, especially since soybean harvest is performed during the dry season.

ANOVA results showed that there was a significant difference between soybean accessions based on vegetative growth measured including plant height, length, and width of terminal leaflets and agronomic characters such as flowering date, TSW, and YLD parameters. Similar results were obtained by Malek et al. [18] and Shrestha et al. [19], who reported that all studied genotypes unveiled highly significant differences among the tested traits and across the environmental conditions tested. This is an indication of the presence of genetic variability among the genotypes used in the investigation. In the same vein, Iqbal et al. [20] reported significant differences in all the investigated traits among the soybean genotypes tested. All tested traits exhibited varying performance across different locations, and even after combining results from all sites, significant differences were observed for almost all traits, suggesting high G×E interactions. We also observed that late flowering favored longer periods of vegetative growth and an increase in vegetative and reproductive parameters.

Morphological traits and YLD components are the factors contributing to YLD variation among accessions. TSW is positively and significantly correlated with plant height at maturity, days to harvest, PW, and NSP; HSW is positively and significantly correlated with TLL and TLW, PL and PW, SL and SWi, and ST. A very strong correlation between ST and HSW could be explained by the fact that the thicker the seeds the heavier the HSW.

PCA is a useful technique that helps determine the relationships between traits. It identifies the combination of traits that contributes most to the total variation. In this study, only the first two principal components, PC1 and PC2, explained almost 60.46% of the total variation. As both principal components had eigenvalues above 1.0 (PC1 = 6.02; PC2 = 3.64), they were considered relevant in explaining the variation between the 30 accessions. Furthermore, only 16 variables were useful in distinguishing the 30 accessions.

The variable correlation circle showed two groups of variables—one group was correlated with the first dimension, representing YLD components, and the other group was correlated with the second dimension, which was more related to plant size, flowering, harvesting date, NPP and NSP, NL, and YLD. A correlation was then deduced between the variables linked to seed size (SL, ST, SWi, PL, and PW), leaflet area (TLL and TLW), and plant size (PHR1 and PHR8). TSW lies between the two dimensions and is positively correlated with them. Therefore, all variables affect TSW.

The PCA biplot analysis showed that the TGm-1511 genotype had the largest seeds and resulted in a very high HSW, followed by TGm-4045. However, genotype TGm-1253 had the highest YLD as the total weight of its TSW seeds was the highest.

A hierarchical classification of the different genotypes shows that Genotypes TGm-1511 and TGm-4045 form a class (Class 5), whereas Genotype TGm-1253 also forms a class (Class 4). This can be explained by the fact that these genotypes showed different characteristics compared with the other genotypes. However, Classes 1 and 2 grouped accessions with the poorest traits.

The success of genetically enhancing crops depends on the genetic diversity and variability that is available. Among the 30 soybean accessions studied, 17 quantitative traits were measured and analyzed, including the PCV and the GCV for all 17 traits. These characteristics showed significant variation. The phenotypic variances were found to be greater than the genotypic variances, and the PCV was greater than the GCV for all the traits studied. This suggests that the phenotypic expression of the traits is influenced by the environment to some extent. The study's findings are similar to those of Malek et al. [18] and Shilpashree et al. [21] in their study on soybeans and Ezin et al. [14, 22, 23] on pumpkin genotypes, elite rice varieties, and cowpea genotypes, respectively.

We observed no difference and very narrow differences between PCV and GCV for some traits including DF, HSW, ST, SL and SWi, days to harvest, PL, and PW. This suggests minimal environmental influence on these traits and a high selection gain. These findings are in agreement with those of many studies on soybean genotypes such as the works of Gohil, Pandya, and Mehta [24]; Tavaud-Pirra et al. [25]; Malek et al. [18]; and Shilpashree et al. [21]. The study also found that some traits have high PCV and GCV, making it possible to improve these genotypes through the selection of desired traits. However, some traits, such as DH and SL, have very low values of PCV and GCV, indicating limited scope for improvement through selection among genotypes.

In plant breeding, heritability is an important parameter to select highly heritable plant traits. The study found low, medium, and high heritability values for the numerous traits studied. Heritability is considered high when it exceeds 50%, medium when it is between 20% and 50%, and low below 20%. The study found that heritability is high in the case of height, DF, and days to harvest. The study's findings are similar to those of other studies conducted by Visscher, Hill, and Wray [26]; Malek et al. [18]; and Shilpashree et al. [21].

The study also found that high heritability combined with high genetic progress could be an indication of additive genetic action. This means that selection based on these parameters would be more reliable. The study found that plant height, YLD per plot, and flowering date have high heritability associated with high genetic progress as a percentage of the mean, indicating the role of additive gene expression for these traits. These findings are similar to those of other studies conducted by Kumar, Kumari, and Kumar [27].

## 5. Conclusion

The study found significant variation among the 17 vegetative and agronomic traits that were measured. Through analyzing the agronomic performance of soybean accessions, the study was able to identify accessions with high YLDs and the best accessions for specific vegetative growth parameters such as plant height, leaf length, and width.

The study's hierarchical classification of the different accessions showed that Class 4 accessions have very good YLDs, whereas Class 5 accessions have large seeds with very high HSWs. Class 3 accessions, however, have good average values for all quantitative traits.

Environment 1 was found to be the best environment for higher YLDs and best-performing traits at the genotype level, notably in terms of leaf area, seed size, plant size at flowering, and YLD.

## Figures and Tables

**Table 1:** Description of test locations.

Code	Environment name	Longitude	Latitude	Pluviometry (mm)	Temperature	Soil type
E1	IITA Benin	2°19′46″E	6°25′3″N	1200–800	28°–30°	Ferralitic soil
E2	Pénéssoulou	1°33′25.1″E	9°13′50.0″N	950	30°–38°	Ferruginous soil

*Note:* Sources: Statistical Yearbook 2015–2016.

**Table 2:** List of accessions used.

Code varieties	Code accessions	Provenance
V1	TGm-109	IITA Ibadan
V2	TGm-1199	IITA Ibadan
V3	TGm-1253	IITA Ibadan
V4	TGm-1347	IITA Ibadan
V5	TGm-1463	IITA Ibadan
V6	TGm-1511	IITA Ibadan
V7	TGm-1552	IITA Ibadan
V8	TGm-1588	IITA Ibadan
V9	TGm-1805	IITA Ibadan
V10	TGm-4034	IITA Ibadan
V11	TGm-4040	IITA Ibadan
V12	TGm-4045	IITA Ibadan
V13	TGm-4105	IITA Ibadan
V14	TGm-4113	IITA Ibadan
V15	TGm-4129	IITA Ibadan
V16	TGm-4167	IITA Ibadan
V17	TGm-4255	IITA Ibadan
V18	TGm-4360	IITA Ibadan
V19	TGm-4524	IITA Ibadan
V20	TGm-4609	IITA Ibadan
V21	TGm-623	IITA Ibadan
V22	TGX 1830-20E	IITA Benin
V23	TGX 1910-14F	IITA Benin
V24	TGX 1951-3F	IITA Benin
V25	TGX 1987-10F	IITA Benin
V26	TGX 1987-62F	IITA Benin
V27	TGX 1910-14 Local	IITA Benin
V28	TGm-1213	IITA Ibadan
V29	TGX-Janguma	IITA Benin
V30	TGX 1910-10 Local	IITA Benin

**Table 3:** Quantitative parameters measured.

Variables	Definition
Number of leaflets (NL)	NL corresponds to the number of leaflets on a soybean leaf. This variable was collected during the period of full flowering
Length and width of the terminal leaflet (TLL and TLW)	TLL and TLW in centimeters are the distances between the edges of the leaflet, passing through the center of the leaflet, in the longest and shortest directions, respectively. These measurements were taken during the period of full flowering
Flowering date (FD)	FD corresponds to the number of days between the sowing date and the date on which at least 50% of the plants have at least one open flower
Plant height at R1 (PHR1)	PHR1 is the height of the plant at the start of the flowering phase. It was measured using a ruler graduated from the base to the tip of the plant
Plant height at R8 (PHR8)	PHR8 is the height of the plant at full maturity
Harvest date (DH)	DH is the number of days between the sowing date and the harvest date
Number of pods per plant (NPP)	NPP is the number of pods present on the plant at harvest
Pod length and width (PL and PW)	These are both in millimeters and were measured after harvest on pods taken at random
Number of seeds per pod (NSP)	The number of seeds per pod was counted after harvest
Seed length, width, and thickness (SL, SWi, and ST)	These data were taken after hulling the pods
Hundred-seed weight (HSW)	HSW represents the weight in grams of one hundred randomly selected seeds
Total seed weight (TSW)	This is the yield for each plot unit
Yield (YLD), leaf area (LS), and seed size (SSi) were determined according to the following formulas	YLD (t/ha) = average seed yield per plant (kg) on a plot unit × plant population per hectare (plant/ha)

**Table 4:** Qualitative parameters.

Qualitative traits	Characteristics	Collection phase
Color of cotyledon	Yellow	At emergence
	Green	

Color of hypocotyl	Green	At first true leaves unfolded
	Purple	

Pubescence	Absent	Flowering
	Present	

Pubescence color	Grey	Flowering
	Brown	
	Dark brown	

Pubescence density	Sparse	Flowering
	Semisparse	
	Normal	
	Dense	

Type of pubescence	Glabrous	Flowering
	Sparse	
	Dense	
	Curly	
	Puberulent	

Petiole presence	Absent	Flowering
	Present	

Leaflet size	Small	Flowering
	Average	
	Big	

Leaflet shape	Narrow lanceolate	Flowering
	Intermediate	
	Wide oval	

Flower color	White	Flowering
	Yellow	
	Red	
	Purple	

Plant: attitude of branches	Erect	Flowering
	From erect to semierect	
	Semierect	
	Semierect to horizontal	
	Horizontal	

Logging	None	At the beginning of fruiting
	Light	
	Moderate	
	Severe	
	Very severe	

Growth type	Determinate	At the beginning of fruiting and at maturity
	Indeterminate	

Pod dehiscence	Not dehiscent	After harvest
	Slightly dehiscent	
	Moderately dehiscent	
	Dehiscent	
	Very dehiscent	

Color of mature pods	Light brown	After harvest
	Dark brown	
	Black	

Integument color	Yellowish white	After shelling the pods
	Yellow	
	Green	
	Light brown	
	Reddish brown	
	Gray	
	Imperfect black	
	Black	

Integument sharpness	Brilliant	After shelling the pods
	Intermediate	
	Dull	

Color of hilum	Yellow	After shelling the pods
	Light brown	
	Brown	
	Green	
	Gray	
	Imperfect black	
	Black	
	Other color	

**Table 5:** Description of quantitative variables.

Variable	Unit	Max	Min	Mean	CV
NL	leaflets	5	3	3.19	7.09
TLL	cm	15.2	4	8.37	26.02
TLW	cm	8.7	2.13	4.66	19.55
DF	days	63	30	49.28	8.92
PHR1	cm	60.6	12.3	32.84	25.98
PHR8	cm	118.5	13	39.13	42.55
DH	days	148	78	114.99	8.75
NPP	pod/plant	636	9	158.13	76.45
NSP	pod/plant	3	2	2.48	17.29
PL	mm	53.06	24.29	34.75	12.85
PW	mm	19.59	5.02	8.38	20.47
SL	mm	9.41	5.53	7.13	8.564
ST	mm	5.98	2.86	4.59	8.50
SWi	mm	7.42	4.56	5.73	6.24
HSW	gram	22.35	5.4	12.36	18.65
TSW	gram	954.3	1.8	101.52	111.05
YLD	t/ha	5.30	0.01	0.56	52.51

*Note:* Length and width of the terminal leaflet (TLL and TLW), plant height at the start of the flowering phase (PHR1), plant height at full maturity (PHR8), leaf area (LS = TLL × TLW), and seed size (SSi = SL × SWi × ST).

Abbreviations: DF, flowering date; DH, harvest date; HSW, hundred-seed weight; NL, number of leaflets; NPP, number of pods per plant; NSP, number of seeds per pod; PL, pod length; PW, pod width; SL, seed length; ST, seed thickness; SWi, seed width; TSW, total seed weight; YLD, yield.

**Table 6:** ANOVA of quantitative variables.

Variables	ENV	REP	BLOC	GEN	GEN: ENV	ENV: REP	REP: BLOC	BLOC: GEN
	df = 1	df = 2	df = 5	df = 27	df = 25	df = 2	df = 10	df = 43
NL	0.03ns	0.02ns	0.23⁣^∗∗∗^	1.61⁣^∗∗∗^	0.13⁣^∗∗∗^	0.06ns	0.02ns	0.07ns
TLL	368.2⁣^∗∗∗^	12.6⁣^∗∗^	7.6⁣^∗∗^	5⁣^∗∗∗^	1.6⁣^∗∗∗^	2.4ns	1ns	1.5ns
TLW	75.9⁣^∗∗∗^	3.88⁣^∗∗^	3.13⁣^∗∗∗^	2.62⁣^∗∗∗^	1.19⁣^∗∗∗^	0.27ns	0.94ns	0.84ns
DF	938⁣^∗∗∗^	1.4ns	8.6ns	231.6⁣^∗∗∗^	48.3⁣^∗∗∗^	3.1ns	32.3⁣^∗∗∗^	21.6⁣^∗∗∗^
PHR1	1498.9⁣^∗∗∗^	38.5ns	174.1⁣^∗∗^	242.2⁣^∗∗∗^	62.4⁣^∗∗∗^	58ns	107.1⁣^∗^	39.7ns
PHR8	11,129⁣^∗∗∗^	145ns	172ns	846⁣^∗∗∗^	293⁣^∗∗∗^	51ns	188ns	135ns
DH	64.3⁣^∗∗^	27.7⁣^∗^	65.9⁣^∗∗∗^	378.3⁣^∗∗∗^	108.7⁣^∗∗∗^	8.4ns	61.6⁣^∗∗∗^	66.3⁣^∗∗∗^
NPP	8417ns	21811ns	91,147⁣^∗∗∗^	19,877⁣^∗∗^	5938⁣^∗∗∗^	49,443⁣^∗∗^	16,280⁣^∗^	12,841⁣^∗^
PL	1146.5⁣^∗∗∗^	25.2⁣^∗^	40.9⁣^∗∗∗^	85.1⁣^∗∗∗^	21.3⁣^∗∗∗^	8.1ns	13.8ns	14.8⁣^∗^
PW	158.04⁣^∗∗∗^	1.26ns	1.92⁣^∗∗^	3.59⁣^∗∗∗^	1.77⁣^∗∗∗^	2.74⁣^∗∗^	2.53⁣^∗∗∗^	3.32⁣^∗∗∗^
NSP	1.1233⁣^∗∗^	0.07ns	0.83⁣^∗∗∗^	0.56⁣^∗∗∗^	0.18⁣^∗∗∗^	0.17ns	0.22⁣^∗^	0.17⁣^∗^
SL	24.48⁣^∗∗∗^	0.28ns	0.42⁣^∗∗^	2.31⁣^∗∗∗^	0.45⁣^∗∗∗^	0.26ns	0.41⁣^∗∗∗^	0.28⁣^∗∗∗^
SWi	0.88⁣^∗∗^	0.84⁣^∗∗∗^	0.41⁣^∗∗^	1.40⁣^∗∗∗^	0.19⁣^∗∗∗^	0.27ns	0.15ns	0.13ns
ST	2.51⁣^∗∗∗^	0.34⁣^∗^	0.33⁣^∗∗^	1.34⁣^∗∗∗^	0.21⁣^∗∗∗^	0.26⁣^∗^	0.32⁣^∗∗∗^	0.16⁣^∗∗^
HSW	223.62⁣^∗∗∗^	6.69⁣^∗^	13.84⁣^∗∗∗^	50.96⁣^∗∗∗^	7.69⁣^∗∗∗^	3.37ns	6.9⁣^∗∗∗^	5.15⁣^∗∗∗^
TSW	604,051⁣^∗∗∗^	21,480⁣^∗^	3657ns	42,416⁣^∗∗∗^	14,932⁣^∗∗∗^	18702ns	4883ns	8900ns
YLD	18,643,535⁣^∗∗∗^	662,957⁣^∗^	112872ns	1,309,148⁣^∗∗∗^	460,851⁣^∗∗∗^	577235ns	150710ns	274693ns

*Note:* Length and width of the terminal leaflet (TLL and TLW), plant height at the start of the flowering phase (PHR1), plant height at full maturity (PHR8), leaf area (LS = TLL × TLW), and seed size (SSi = SL × SWi × ST).

Abbreviations: df, degree of freedom; DF, flowering date; DH, harvest date; HSW, hundred-seed weight; NL, number of leaflets; NPP, number of pods per plant; ns, nonsignificant; NSP, number of seeds per pod; PL, pod length; PW, pod width; SL, seed length; ST, seed thickness; SWi, seed width; TSW, total seed weight; YLD, yield.

⁣^∗∗∗^*p* < 0.001.

⁣^∗∗^*p* < 0.01.

⁣^∗^*p* < 0.05.

**Table 7:** ANOVA for morphological parameters.

Accession	NL	TLL	TLW	PH R1	PH R8	DF
V1	3 ± 0.0d	9 ± 1.89bc	4.19 ± 0.18cdef	40.76 ± 12.55abc	44.82 ± 4.82abcde	54.80 ± 1.30a
V2	3 ± 0.0d	9.84 ± 0.78ab	4.16 ± 0.49cdef	35.10 ± 1.87abcd	66.95 ± 25.12a	55.75 ± 1.5a
V3	3 ± 0.0d	8.75 ± 2.11bc	5.10 ± 1.43bcd	44.10 ± 10.87a	51.340 ± 5.44abcd	43.16 ± 12.4de
V4	3.33 ± 0.82c	7.71 ± 2.78bc	4.56 ± 1.59bcde	30.60 ± 12.65d	51.14 ± 30.85abcd	56.66 ± 1.86a
V5	3 ± 0.0d	6.85 ± 1.76bc	2.93 ± 0.63f	29.58 ± 6.77d	44.48 ± 24.72bcde	39 ± 4.51e
V6	3 ± 0.0d	12.95 ± 2.63a	7.22 ± 1.78a	24.91 ± 1.72def	23.61 ± 5.64ef	40 ± 0.00e
V7	3 ± 0.0d	7.54 ± 2.49bc	4.10 ± 1.47cdef	24.19 ± 2.97def	25.02 ± 3.73ef	51.50 ± 2.95abc
V8	3.67 ± 1.15b	9.89 ± 3.02ab	3.93 ± 0.75def	39.18 ± 10.60abcd	42.43 ± 13.30bcde	42.66 ± 8.08de
V9	3 ± 0.0d	7.72 ± 1.64bc	4.09 ± 0.84cdef	30.52 ± 7.25d	35.98 ± 9.69def	46.83 ± 7.76cd
V10	3 ± 0.0d	7.30 ± 2.78bc	4.54 ± 1.64bcde	33.05 ± 10.23bcd	41.16 ± 4.30abcde	54.00 ± 2ab
V11	3 ± 0.0d	8.14 ± 1.85bc	4.61 ± 1.22bcde	19.22 ± 3.45ef	17.26 ± 2.43f	42.80 ± 3.89de
V12	3 ± 0.0d	9.17 ± 1.85b	4.71 ± 1.32bcde	24.12 ± 4.11def	25.08 ± 8.72ef	40 ± 2.19e
V13	3 ± 0.0d	9.17 ± 2.53b	4.99 ± 0.94bcde	24 ± 3.98def	25.90 ± 5.48ef	55.20 ± 4.55a
V14	3 ± 0.0d	9.73 ± 1.98b	5.29 ± 1.26bcd	34.73 ± 8.21abcd	63.68 ± 35.15ab	55.33 ± 1.36a
V15	3 ± 0.0d	9.46 ± 2.53bc	5.84 ± 1.42ab	37.58 ± 7.78abcd	41.92 ± 10.09cde	54 ± 0.70ab
V16	3 ± 0.0d	7.83 ± 2.81bc	3.97 ± 1.29cdef	28 ± 12.76de	34.20 ± 14.49def	39.25 ± 8.38e
V17	3 ± 0.0d	7.82 ± 2.58bc	5.06 ± 1.79bcd	31.76 ± 9.53cd	46.84 ± 23.59abcd	41 ± 3.94e
V18	3 ± 0.0d	7.46 ± 1.94bc	3.58 ± 0.95ef	35.58 ± 8.55abcd	62.26 ± 35.32abc	53.83 ± 2.71ab
V19	3 ± 0.0d	8.10 ± 1.80bc	4.37 ± 0.63cdef	32.44 ± 5.99bcd	34.69 ± 7.63def	54.50 ± 0.76a
V20	3 ± 0.0d	6.26 ± 1.10c	3.96 ± 0.6cdef	16 ± 4.78f	15.55 ± 1.05f	32 ± 4f
V21	3 ± 0.0d	6.96 ± 0.66bc	3.93 ± 0.95def	29.93 ± 11.46d	39.58 ± 19.23de	55 ± 3.79a
V22	5 ± 0.0a	8.13 ± 1.06bc	3.93 ± 1.24def	33.86 ± 10.07bcd	32.88 ± 11.14def	54.16 ± 2.86a
V23	3 ± 0.0d	9.32 ± 1.25bc	5.45 ± 1bc	37.48 ± 5.89abcd	37.85 ± 6.49def	53.16 ± 1.33ab
V24	5 ± 0.0a	8.38 ± 2.22bc	4.76 ± 1.07bcde	41.56 ± 9.69ab	42.40 ± 9.93cde	49 ± 2.45bc
V25	3 ± 0.0d	8.25 ± 1.79bc	5.23 ± 1.13bcd	34.88 ± 6.21abcd	37.14 ± 5.36def	53.16 ± 2.93ab
V26	3 ± 0.0d	8.69 ± 2.21bc	5.20 ± 1.38bcd	41.25 ± 10.64abc	41.87 ± 14.57de	50.83 ± 3.25abc
V27	3 ± 0.0d	8.71 ± 1.98bc	4.98 ± 1.1bcde	33.02 ± 5.42bcd	33.75 ± 8.50def	55.16 ± 3.97a
V28	3 ± 0.0d	8.50 ± 2.65bc	4.50 ± 1.13bcdef	40 ± 11.45abcd	38 ± 6.54def	59.00 ± 7.98a

*Note:* The mean survival of the same alphabetical letters is not significantly different at the 0.05 threshold according to LSD. Length and width of the terminal leaflet (TLL and TLW), plant height at the start of the flowering phase (PHR1), plant height at full maturity (PHR8), leaf area (LS = TLL × TLW), and seed size (SSi = SL × SWi × ST).

Abbreviations: DF, flowering date; DH, harvest date; HSW, hundred-seed weight; NL, number of leaflets; NPP, number of pods per plant; NSP, number of seeds per pod; PL, pod length; PW, pod width; SL, seed length; ST, seed thickness; SWi, seed width; TSW, total seed weight; YLD, yield.

**Table 8:** ANOVA for yield parameters.

Accession	NPP	PL	PW	NSP	SL
V1	273.80 ± 179.48a	29.33 ± 9.31hi	7.75 ± 2.42c	2.4 ± 0.87bcdef	6.15 ± 0.45k
V2	194 ± 39.99abcdef	44.19 ± 12.75ab	10.50 ± 2.91a	3 ± 0.87a	8.82 ± 0.27a
V3	230.80 ± 131.61abcd	33.67 ± 9.74cdefgh	7.47 ± 2.16c	2.80 ± 0.76abc	6.36 ± 0.26ijk
V4	117.80 ± 89.05bcdef	38.39 ± 9.49bc	8.68 ± 2.18abc	3 ± 0.29a	7.15 ± 0.40defgh
V5	110 ± 72.70cdef	33.20 ± 8.24cdefgh	7.66 ± 1.41c	3 ± 0.87a	7.78 ± 0.44bcd
V6	134.33 ± 33.50abcdef	46.20 ± 1.61a	10.52 ± 0.14a	2.33 ± 0.29cdef	9.04 ± 0.32a
V7	158 ± 35.68abcdef	30.22 ± 0.60ghi	9.82 ± 3.72ab	2.17 ± 0.29ef	6.71 ± 0.38fghijk
V8	173.33 ± 45.03abcdef	44.31 ± 20.33ab	9.06 ± 4.37abc	2.63 ± 1.26abcde	7.72 ± 1.24bcde
V9	190.50 ± 52.89abcdef	27.43 ± 1.37i	7.19 ± 0.13c	2.17 ± 0.29ef	6.69 ± 0.51fghijk
V10	87.20 ± 53.05def	37.19 ± 2.41cde	7.87 ± 0.39bc	2.5 ± 0.5abcdef	7.20 ± 0.90defg
V11	54.40 ± 27.97f	34.95 ± 9.77cdefg	9.10 ± 2.48abc	2.20 ± 0.76def	6.95 ± 0.66efghij
V12	82.17 ± 56.65ef	42.54 ± 3.41ab	9.94 ± 0.64a	2.17 ± 0.29ef	8.24 ± 0.57ab
V13	79 ± 35.87ef	35.16 ± 7.45cdefg	8.54 ± 1.79abc	2 ± 0.58f	7.35 ± 0.54cdef
V14	180.33 ± 49.07abcdef	34.66 ± 0.44cdefgh	8.29 ± 0.39abc	2.33 ± 0.29cdef	7.15 ± 0.32defgh
V15	90.40 ± 31.46def	38.08 ± 10bcd	10.01 ± 2.93a	2.4 ± 0.5bcdef	7.10 ± 0.55defghi
V16	67 ± 27.00ef	35.32 ± 10.62cdefg	8.02 ± 2.58abc	2 ± 0.58f	7.71 ± 1.27bcde
V17	125 ± 78.02bcdef	33.70 ± 3.34cdefgh	8.29 ± 0.07abc	2.5 ± 0.5abcdef	7.21 ± 0.53defg
V18	251.20 ± 80.82abc	32.53 ± 10.06cdefghi	7.63 ± 1.69c	3 ± 0.87a	6.42 ± 0.43hjk
V19	184.5 ± 168.32abcdef	31.15 ± 1.37fghi	7.21 ± 0.37c	2.67 ± 0.29abcd	6.24 ± 0.26jk
V20	49 ± 44.03f	31.55 ± 9.20fghi	8.04 ± 2.14abc	2.5 ± 0.76abcdef	6.51 ± 0.30ghijk
V21	100 ± 59.63def	34.02 ± 7.59cdefgh	8.38 ± 2.26abc	2.21 ± 0.76def	6.12 ± 2.02k
V22	164.17 ± 104.22abcdef	32.42 ± 0.81efghi	7.81 ± 0.06c	2.17 ± 0.29ef	7.36 ± 0.89cdef
V23	250.33 ± 110.27ab	35.28 ± 0.82cdefg	8.49 ± 0.1abc	2.5 ± 0.02abcdef	7.54 ± 0.67cde
V24	261.83 ± 108.61ab	37.38 ± 1.78cde	8.56 ± 0.50abc	2.33 ± 0.29cdef	7.92 ± 0.75bc
V25	140.5 ± 84.77abcdef	31.95 ± 1.31fghi	7.86 ± 0.55bc	2.17 ± 0.29ef	6.41 ± 0.59ijk
V26	217.83 ± 147.52abcde	30.46 ± 2.53fghi	7.53 ± 0.29c	2.83 ± 0.29ab	6.78 ± 0.42fghijk
V27	186.33 ± 100.79abcdef	35.39 ± 0.79cdef	7.93 ± 0.10bc	3 ± 0.6a	7.21 ± 0.81defg
V28	216 ± 145.06abcdef	27.85 ± 2.15hi	6.64 ± 0.67c	2 ± 0.34f	6.18 ± 0.61jk

**Accessions**	**SWi**	**ST**	**HSW**	**TSW**	**YLD**

V1	4.86 ± 0.18j	4.09 ± 0.65jk	7.30 ± 0.43l	114.76 ± 52.80a	637.56 ± 152b
V2	6.35 ± 0.22b	4.62 ± 0.70efgh	15.74 ± 2.90bcd	596.08 ± 99.95a	3311.59 ± 222a
V3	5.50 ± 0.21efgh	4.48 ± 0.18fghij	9.63 ± 1.02hijkl	63.27 ± 27.46b	351.50 ± 72b
V4	5.17 ± 0.31ghij	4.10 ± 0.12ijk	10.97 ± 1.03fghij	97.44 ± 83.03b	541.37 ± 173b
V5	4.82 ± 0.11j	3.07 ± 0.23l	8.15 ± 0.62jkl	63.15 ± 36.12b	350.88 ± 103b
V6	6.98 ± 0.42a	5.65 ± 0.28a	21.29 ± 1.71a	90.67 ± 4.70b	503.76 ± 52b
V7	5.66 ± 0.18def	4.74 ± 0.18defg	10.83 ± 0.43ghij	54.23 ± 2.40b	301.28 ± 67b
V8	5.99 ± 0.8bcde	5 ± 0.65bcdef	15.95 ± 7.55bcd	161.71 ± 71.86b	898.39 ± 79b
V9	5.12 ± 0.27hij	3.98 ± 0.53k	8.778 ± 2.21jkl	52.42 ± 16.02b	291.20 ± 32b
V10	5.63 ± 0.31ef	4.67 ± 0.43defg	12.70 ± 1.62efg	83.61 ± 44.96b	464.51 ± 166b
V11	6.18 ± 0.33bc	4.93 ± 0.33cdef	12.68 ± 2.87efg	33.52 ± 7.43b	186.22 ± 76b
V12	6.89 ± 0.35a	5.44 ± 0.45ab	18.17 ± 1.87ab	82.76 ± 48.74b	459.81 ± 126b
V13	5.81 ± 0.51cdef	4.58 ± 0.54efghi	13.98 ± 1.89cde	30.81 ± 10.08b	171.19 ± 61b
V14	6.08 ± 0.30bc	5.07 ± 0.13bcd	13.41 ± 1.25defg	96.80 ± 63.09b	537.77 ± 137b
V15	6.10 ± 0.50bc	5.24 ± 0.52abc	17.50 ± 3.02b	93.46 ± 32.57b	519.27 ± 190b
V16	5.84 ± 0.61cdef	4.17 ± 0.65hijk	12.71 ± 4.67defg	37.13 ± 25.96b	206.30 ± 56b
V17	6.13 ± 0.31bc	4.58 ± 0.30fghi	13.05 ± 2.97defg	94.20 ± 40.13b	523.31 ± 137b
V18	4.98 ± 0.22ij	3.83 ± 0.22k	7.80 ± 1.25kl	139.90 ± 99.72b	777.25 ± 103b
V19	5.18 ± 0.21ghij	4.20 ± 0.21hijk	8.75 ± 1.39jkl	71.06 ± 55.28b	394.77 ± 133b
V20	5.86 ± 0.06cde	4.92 ± 0.20cdef	10.60 ± 1.62ghijk	17.77 ± 9.10b	98.76 ± 43b
V21	5.08 ± 0.32hij	4.27 ± 0.15ghijk	8.90 ± 0.74ijkl	49.38 ± 16.49b	329.19 ± 103b
V22	5.57 ± 0.41efg	4.62 ± 0.43efgh	13.30 ± 1.89defg	113.63 ± 39.65b	631.26 ± 159b
V23	6.06 ± 0.36bcd	4.70 ± 0.20defg	13.49 ± 1.87def	122.02 ± 44.02b	677.89 ± 111b
V24	6.14 ± 0.26bc	5.04 ± 0.31bcde	16.49 ± 4.67bc	134.74 ± 40.13b	748.54 ± 117b
V25	5.39 ± 0.53fghi	4.58 ± 0.40fghi	11.58 ± 1.62efghi	69.06 ± 32.10b	383.64 ± 143b
V26	5.88 ± 0.31cde	4.97 ± 0.33cdef	12.21 ± 1.43efgh	122.52 ± 51.90b	680.67 ± 115b
V27	5.95 ± 0.44bcde	4.68 ± 0.45defg	13.25 ± 2.91defg	141.36 ± 88.92b	785.30 ± 153b
V28	5.33 ± 0.63fghij	3.56 ± 0.35kl	7.60 ± 0.41kl	20.34 ± 8.62b	113.00 ± 85b

*Note:* The mean survival of the same alphabetical letters is not significantly different at the 0.05 threshold according to LSD. Length and width of the terminal leaflet (TLL and TLW), plant height at the start of the flowering phase (PHR1), plant height at full maturity (PHR8), leaf area (LS = TLL × TLW), and seed size (SSi = SL × SWi × ST).

Abbreviations: DF, flowering date; DH, harvest date; HSW, hundred-seed weight; NL, number of leaflets; NPP, number of pods per plant; NSP, number of seeds per pod; PL, pod length; PW, pod width; SL, seed length; ST, seed thickness; SWi, seed width; TSW, total seed weight; YLD, yield.

**Table 9:** Eigenvalues of quantitative variables.

	Eigenvalue	% Variance inertia	% Cumulative variance
Comp1	6.02569239	37.6605775	37.66058
Comp2	3.64921813	22.8076133	60.46819
Comp3	1.74893166	10.9308229	71.39901
Comp4	1.27411908	7.9632442	79.36226
Comp5	1.01099451	6.3187157	85.68097
Comp6	0.63314204	3.9571377	89.63811
Comp7	0.47387268	2.9617043	92.59982
Comp8	0.34851183	2.1781990	94.77801
Comp9	0.24766240	1.5478900	96.32590
Comp10	0.19014492	1.1884058	97.51431
Comp11	0.13287614	0.8304758	98.34479
Comp12	0.11236957	0.7023098	99.04710
Comp13	0.07280315	0.4550197	99.50212
Comp14	0.04020262	0.2512663	99.75338
Comp15	0.02883886	0.1802429	99.93362
Comp16	0.01062002	0.0663751	100.00000

**Table 10:** Coordinates of quantitative variables in the two dimensions.

Variables	Dim. 1	Dim. 2
NL	0.1090556	0.24539918
TLL	0.6927466	0.33200667
TLW	0.6074149	0.04894688
DF	−0.2274988	0.62628384
PHR1	−0.3416530	0.74500887
PHR8	−0.2583721	0.78361256
DH	0.1475043	0.76166189
NPP	−0.3086289	0.68566184
PL	0.8728721	0.17511500
PW	0.8695991	0.02612490
NSP	−0.1062927	0.42139471
SL	0.8279638	0.22394758
SWi	0.9405411	−0.05790817
ST	0.8234340	−0.15330110
HSW	0.9667483	0.03706608
TSW	0.3170274	0.75992867

**Table 11:** Description of each class by variables and modalities.

	Class 1	Class 2	Class 3	Class 4	Class 5
DF	0.776	−2.29	1.12	1.01	−2.02
DH	−0.65	−3.51	2.02	2.32	−0.906
HSW	−3.88	−0.184	1.81	0.667	3.23
NPP	1.63	−2.02	−0.102	0.404	−1.03
PHR1	153	−3	0.862	0.397	−1.75
PHR8	1	−2.81	0.822	1.61	−1.76
PL	−3.14	−0.373	0.784	1.97	3.12
PW	−3.26	0.168	0.788	1.98	2.78
SL	−3.19	−0.8	1.18	2.12	2.78
ST	−3.76	0.999	1.81	−0.405	2.6
SWi	−4.04	0.821	1.36	1.03	3.12
TLL	−1.49	−1.42	0.0908	1.04	3.21
TLW	−1.77	−0.402	0.809	−0.608	2.48
TSW	−1.26	−1.36	0.24	4.51	−0.076

**Table 12:** Ranking of the best genotypes in Environment 1.

Rank	ENV	LS	PHR1	NPP	NSP	PL	SSi	HSW	YLD
1	E1	V6	V3	V1	V2	V8	V6	V6	V2
2	E1	V15	V24	V18	V4	V6	V12	V8	V27
3	E1	V14	V1	V8	V5	V2	V8	V15	V8
4	E1	V17	V26	V7	V8	V12	V2	V24	V18
5	E1	V12	V8	V2	V10	V16	V24	V12	V22
6	E1	V3	V10	V24	V18	V10	V16	V2	V10
7	E1	V10	V22	V22	V19	V15	V27	V16	V24
8	E1	V26	V17	V9	V27	V27	V15	V27	V26
9	E1	V23	V18	V14	V3	V24	V23	V22	V1
10	E1	V4	V15	V3	V26	V4	V22	V13	V23
11	E1	V25	V21	V17	V6	V21	V17	V23	V17
12	E1	V27	V14	V27	V15	V17	V14	V17	V4
13	E1	V13	V23	V23	V17	V23	V11	V10	V14
14	E1	V24	V25	V26	V20	V11	V26	V14	V12
15	E1	V7	V27	V10	V21	V13	V10	V26	V15
16	E1	V8	V4	V4	V1	V14	V13	V11	V25
17	E1	V22	V2	V19	V7	V22	V20	V25	V19
18	E1	V1	V5	V21	V9	V19	V7	V4	V7
19	E1	V19	V9	V25	V11	V5	V25	V7	V5
20	E1	V2	V19	V6	V12	V3	V9	V20	V21
21	E1	V16	V16	V5	V13	V25	V3	V9	V6
22	E1	V21	V7	V13	V14	V18	V4	V3	V9
23	E1	V11	V12	V15	V16	V26	V1	V21	V16
24	E1	V9	V6	V12	V22	V7	V19	V5	V3
25	E1	V18	V13	V16	V23	V20	V18	V19	V11
26	E1	V5	V11	V11	V24	V1	V21	V18	V13
27	E1	V20	V20	V20	V25	V9	V5	V1	V20

**Table 13:** Ranking of the best genotypes in Environment 2.

Rank	ENV	LS	PHR1	NPP	NSP	PL	SSi	HSW	YLD
1	E2	V23	V3	V23	V1	V2	V12	V12	V24
2	E2	V28	V28	V24	V2	V12	V14	V15	V23
3	E2	V2	V23	V3	V3	V4	V24	V24	V3
4	E2	V14	V15	V26	V4	V24	V15	V14	V26
5	E2	V11	V26	V18	V5	V13	V11	V13	V19
6	E2	V3	V2	V28	V18	V23	V13	V23	V14
7	E2	V13	V24	V19	V20	V10	V2	V11	V27
8	E2	V27	V19	V27	V23	V14	V23	V2	V2
9	E2	V20	V25	V9	V26	V15	V17	V17	V18
10	E2	V26	V14	V1	V27	V20	V26	V20	V25
11	E2	V25	V18	V14	V14	V11	V7	V22	V9
12	E2	V15	V4	V25	V17	V3	V20	V26	V1
13	E2	V19	V1	V2	V24	V18	V4	V10	V20
14	E2	V1	V8	V20	V11	V5	V10	V27	V28
15	E2	V24	V27	V22	V15	V27	V3	V7	V12
16	E2	V12	V5	V7	V7	V25	V27	V25	V22
17	E2	V16	V22	V5	V9	V17	V22	V4	V17
18	E2	V9	V9	V15	V10	V1	V21	V3	V5
19	E2	V22	V16	V16	V12	V22	V25	V21	V15
20	E2	V8	V10	V17	V19	V21	V19	V19	V11
21	E2	V18	V13	V12	V22	V26	V8	V16	V7
22	E2	V4	V17	V11	V25	V28	V16	V28	V4
23	E2	V17	V20	V4	V8	V8	V28	V5	V21
24	E2	V5	V7	V21	V13	V19	V18	V9	V10
25	E2	V7	V12	V13	V16	V7	V5	V8	V13
26	E2	V21	V21	V8	V21	V16	V9	V18	V16
27	E2	V10	V11	V10	V28	V9	V1	V1	V8

**Table 14:** Performance of genotypes and their environments.

Parameter	Min	Max	MinENV	MaxENV	MinGEN	MaxGEN
LS	8.6 (V16 in E2)	132.24 (V6 in E1)	E2 (27.5)	E1 (53.51)	V5 (22.53)	V6 (96.73)
PHR1	12.3 (V20 in E1)	60.6 (V1 in E1)	E2 (29.54)	E1 (35.4)	V20 (16.01)	V3 (47.52)
NPP	9 (V12 in E2)	636 (V26 in E2)	E2 (148.86)	E1 (164.4)	V20 (49)	V1 (273.8)
NSP	2 (V1 in E1)	3 (V2 in E1)	E1 (2.4)	E2 (2.57)	V13 (2)	V2 (3)
PL	24.29 (V17 in E2)	53.06 (V8 in E1)	E2 (31.53)	E1 (37.26)	V9 (27.43)	V6 (46.2)
SSi	87.86 (V9 in E2)	416.97 (V6 in E1)	E2 (170.63)	E1 (210.01)	V5 (115.3)	V6 (358.7)
HSW	5.4 (V9 in E2)	22.35 (V6 in E1)	E2 (10.97)	E1 (13.46)	V1 (7.3)	V6 (21.29)
YLD	0.01(V19 in E2)	5.3 (V2 in E1)	E2 (0.15)	E1 (0.89)	V20 (0.1)	V2 (3.31)

**Table 15:** Estimation of genetic parameters for soybean accessions studied.

Parameter	Mean	GV	PV	*H* ^2^ (%)	GCV (%)	PCV (%)	GA	GA (%)
NL	3.18	0.28	0.33	84.33	16.54	18.01	0.99	31.29
TLL	9.79	1.10	2.89	37.98	10.70	17.36	1.33	13.58
TLW	5.24	0.87	1.51	57.32	17.76	23.46	1.45	27.70
DF	47.23	73.04	73.04	100	18.10	18.10	17.61	37.28
PHR1	35.32	66.71	120.06	55.56	23.13	31.02	12.54	35.51
PHR8	47.04	305.77	483.78	63.20	37.17	46.76	28.64	60.88
NPBM	6.85	2.74	4.93	55.62	24.17	32.41	2.54	37.13
DH	115.65	133.70	133.70	100	10	10	23.82	20.60
NPP	162.33	1835.30	9063	20.25	26.40	58.65	39.71	24.46
PL	37.24	24.39	32.70	74.60	13.26	15.36	8.79	23.60
PW	9.32	0.64	1.15	55.93	8.60	11.50	1.23	13.26
NSP	2.39	0.14	0.24	56.72	15.48	20.55	0.57	24.01
SL	7.49	0.66	0.76	85.95	10.80	11.65	1.55	20.63
SWi	5.80	0.33	0.42	78.29	9.89	11.17	1.04	18.02
ST	4.71	0.28	0.35	78.23	11.14	12.59	0.96	20.29
HSW	13.48	14.05	15.64	89.84	27.81	29.34	7.32	54.29
TSW	155.88	16,353.83	23,782.09	6877	82.04	98.93	218.45	140.14

**Table 16:** Variation in qualitative traits of soybean accessions.

Qualitative traits	Level of traits	Number of accessions	Frequency (%)
Hypocotyl color	Green	6	22.22
	Purple	21	77.78

Pubescence color	Gray	7	25.93
	Light brown	14	51.85
	Dark brown	6	22.22

Pubescence density	Sparse	5	18.52
	Semisparse	6	22.22
	Normal	11	40.74
	Dense	5	18.52

Pubescence type	Trained	8	29.63
	Semiappressed	12	44.44
	Appressed	6	22.22
	Curly	1	3.70

Leaflet size	Small	25	92.59
	Average	2	7.41

Leaflet shape	Narrow lanceolate	8	29.63
	Intermediate	4	14.81
	Wide oval	15	55.56

Flower color	Blanc	4	14.81
	Purple	23	85.19

Plant habit	Trained	6	22.22
	Erect semierect	6	22.22
	Half erect	13	48.15
	Semierect to horizontal	2	7.41

Verse	None	12	44.44
	Light	9	33.33
	Moderate	2	7.41
	Severe	3	11.11
	Very severe	1	3.70

Growth type	Determinate	16	59.26
	Indeterminate	11	40.74

Pod dehiscence	Not dehiscent	11	40.74
	Slightly dehiscent	13	48.15
	Dehiscent	1	3.70
	Very dehiscent	2	7.41

Color of mature pods	Light brown	16	59.26
	Dark brown	10	37.04
	Black	1	3.70

Integument color	Yellowish white	2	7.41
	Light brown	20	74.07
	Reddish brown	2	7.41
	Imperfect black	1	3.70
	Black	2	7.41

Tegument glossiness	Brilliant	5	18.52
	Intermediate	9	33.33
	Dull	13	48.15

Color of hilum	Light brown	5	18.52
	Brown	6	22.22
	Gray	2	7.40
	Imperfect black	10	37.04
	Black	4	14.81

## Data Availability

The datasets used and analyzed during the current study are available from the corresponding author upon reasonable request.
